# Trichloroethylene Hypersensitivity Syndrome Is Potentially Mediated through Its Metabolite Chloral Hydrate

**DOI:** 10.1371/journal.pone.0127101

**Published:** 2015-05-28

**Authors:** Yongshun Huang, Lihua Xia, Qifeng Wu, Zifang Zeng, Zhenlie Huang, Shanyu Zhou, Jiachun Jin, Hanlin Huang

**Affiliations:** 1 Department of Occupational Health and Occupational Medicine, School of Public Health and Tropical Medicine, Southern Medical University, Guangzhou, Guangdong, 510505, PR China; 2 Department of Occupational Medicine, Guangdong Province Hospital for Occupational Disease Prevention and Treatment, Guangzhou, Guangdong, 510300, PR China; Harvard Medical School, UNITED STATES

## Abstract

**Background:**

We documented previously the entity of trichloroethylene (TCE) hypersensitivity syndrome (THS) in occupational workers.

**Objectives:**

To identify the culprit causative compound, determine the type of hypersensitivity of THS, and establish a screening test for subjects at risk of THS.

**Methods:**

TCE and its main metabolites chloral hydrate (CH), trichloroethanol (TCOH) and trichloroacetic acid (TCA) were used as allergens at different concentrations in skin patch tests. The study included 19 case subjects diagnosed with occupational THS, 22 control healthy workers exposed to TCE (exposure >12 weeks), and 20 validation new workers exposed to TCE for <12 weeks free of THS. All subjects were followed-up for 12 weeks after the patch test.

**Results:**

The highest patch test positive rate in subjects with THS was for CH, followed by TCOH, TCA and TCE. The CH patch test positive rate was 100% irrespective of CH concentrations (15%, 10% and 5%). The TCOH patch test positive rate was concentration-dependent (89.5%, 73.7% and 52.6% for 5%, 0.5% and 0.05%, respectively). Lower patch test positive rates were noted for TCA and TCE. All patch tests (including four allergens) were all negative in each of the 22 control subjects. None of the subjects of the validation group had a positive 15% CH patch test.

**Conclusions:**

Chloral hydrate seems to be the culprit causative compound of THS and type IV seems to be the major type of hypersensitivity of THS. The CH patch test could be potentially useful for screening workers at risk of THS.

## Introduction

Trichloroethylene (TCE) is a chlorinated solvent used extensively in industrial operations involving metal cleaning and degreasing. Workers exposed to TCE present with trichloroethylene hypersensitivity syndrome (THS, or Occupational Medicamentosa-like Dermatitis Induced by Trichloroethylene, according to the Chinese National Legal Occupational Disease List), which is characterized by fever, generalized rash, liver dysfunction and superficial lymphadenopathy. Cases with THS have so far been reported from China [1,2], and so far, 394 cases have been diagnosed in the Guangdong Province in Southern China [3].

Based on the associated clinical features, frequent relapse, low incidence but high mortality, THS has become an important occupational health issue in Asia. Over the last 20 years, worldwide studies on the etiology, pathogenesis, occupational epidemiology and countermeasures of THS have been published [2,4–8]. Studies on patients with THS have indicated that it is an occupational immunological disease, with a latency extending from 5 to 40 days, but no longer than 80 days [7]. In a series of studies from our group in collaboration with Japanese researchers, we described four dermatological manifestations of THS; exfoliative dermatitis (ED), erythema multiforme (EM), Stevens-Johnson syndrome (SJS) and toxic epidermal necrolysis (TEN) [2,4,9]. The pathogenic mechanisms of the four phenotypes are not fully understood. The major type of TCE-induced hypersensitivity remains the subject of debate, THS is probably a type IV hypersensitivity [6], while types II and III hypersensitivity [10] also seem to be related to THS. Despite extensive research, critical questions remain unanswered regarding whether THS is caused by TCE itself or its metabolites, and the best tool to be used for screening and protecting susceptible populations.

Some patients with THS experienced recurrences after re-exposure to TCE [11–13], suggesting a clear relationship between TCE exposure and occurrence of the hypersensitivity syndrome. We previously investigated four cases with THS by the patch test; the result showed that metabolites of TCE might be the culprit causative compound [14]. Other patch tests performed in THS demonstrated a positive reaction for TCE and its metabolites. Positive results are more frequently seen with compounds of TCE than parent chemical. These results suggest that the patients are allergic to both TCE and its main metabolites. Based on the positive patch test results, we hypothesized that the culprit compound responsible for THS is not TCE itself, but rather its metabolites. The present case—control follow-up study was designed to determine the culprit compound causing THS and the type of hypersensitivity in THS, using the patch test.

## Material and Methods

### Study subjects

A total of 61 subjects were enrolled in this study. They were divided into three groups. The case group included 19 rehabilitation workers with THS who had been admitted to the Guangdong Province Hospital for Occupational Disease Prevention and Treatment (GDHOD). THS was diagnosed by three occupational dermatologists at the GDHOD, according to The Chinese National Diagnostic Criteria for Occupational Medicamentosa-like Dermatitis Induced by trichloroethylene (GBZ185-2006) (Ministry of Health, China, 2006) (S1 Text). The rash type in these patients included the ED type (n = 17, 89.5%), and EM type (n = 2, 10.5%) (S1 Fig). The subjects had history of industrial exposure to TCE and presented with clinical manifestations of THS including fever, generalized rash, liver dysfunction and superficial lymphadenopathy. The diagnosis of THS was based on the appearance of symptoms within the first 12 weeks of exposure to TCE. The patients were treated with glucocorticoids, and the criteria for response to therapy included the disappearance of rash, resolution of fever, normalization of liver function tests, and lack of relapse within 2 weeks of cessation of glucocorticoids. The control group comprised 22 healthy workers who were exposed to TCE for more than 12 weeks but did not develop THS. Workers with current or past history of skin lesions were excluded from the study. The validation group comprised 20 new workers exposed to TCE within the first 12 weeks of commencement of work, and was followed-up for 12 weeks after the skin patch test. Workers with current or past history of skin lesions were also excluded from the study. None of the study subjects had been on treatment with corticosteroids, other immunosuppressants, or antibiotics.

The study protocol was conducted according to the principles of the Declaration of Helsinki and was approved by the Scientific and Medical Ethical Committee of GDHOD. All the subjects gave written informed consent before inclusion in the study.

### Preparation of allergens

TCE (Sigma-Aldrich, UK, purity ≥99.5%), chloral hydrate (CH) (Riedel-de Haen, Germany, purity ≥99.5%) and trichloroethanol (TCOH) (91120 Fluka, Sigma-Aldrich, purity ≥98.0%) were mixed in olive oil (Sigma-Aldrich). Trichloroacetic acid (TCA) (Tianjin Chemical Reagent Second Factory, Tianjin, China, purity ≥99.5%) was mixed with saline to prepare different concentrations of the allergen. The concentrations used in the case and control groups were based on our previous trial test and previous studies by other investigators [6,11,13,15–17], and included 50%, 25%, 10% and 5% concentrations for TCE; 15%, 10% and 5% concentrations for CH; 5%, 0.5% and 0.05% concentrations for TCOH; and 5% and 0.5% concentrations for TCA. The highest concentration of allergen that induced the highest positive rate in the patch test was selected as allergen in the validation group.

### The skin patch test

Each well of the patch test apparatus (Yida Medical Equipment, Hebei, China) was filled with 25 μl allergen in olive oil or saline, and numbered before applying the apparatus to the back of the subject. The edge of the apparatus was fixed to the skin with 3M micropore permeable medical tape. The subjects were asked to refrain from taking a shower during the duration of the skin patch test (48 hours). The patch was removed after 48 hr and the skin reaction was recorded in the first 0.5 h, 24 h and 48 h after the removal of the patch, and filed according to the International Contact Dermatitis Research Group (ICDRG) by two professional dermatologists at GDHOD.

### Statistical analysis

Variables with normal distribution were expressed as mean±SD, while parameters with skewed distribution were expressed as median [M(P_25_, P_75_)]. The Kruskal-Wallis H Test was used to compare data of several groups. Pearson χ^2^ test was used for comparison of ratios. Two-sided probability (*p*) value <0.05 was considered statistically significant. Statistical analyses were performed using the SPSS 13.0 software (SAS Institute Inc., Cary, NC).

## Results

### Characteristics of subjects

Subjects of the case group included 15 males (79%) and 4 females (21%), aged between 19 to 55 years. Ethnically, they included 16 Han Chinese and 3 minorities, including Hmong, Zhuang and Gelao ethnic groups. The average duration of exposure to TCE was 34 (range, 31–43) days. The time-weighted average airborne concentration of TCE at the work environment was 95.1 (MP_25_, 62.5, MP_75_, 306.4 mg/m^3^, range, 1.0–440.9 mg/m^3^). Urinary TCA level was measured in all 19 subjects of the case group after admission to the hospital. The results were below the detection threshold (3.0 mg/L) in 7 subjects, and 3.0–122.0 mg/L in the other 12 cases. Subjects of the case groups were treated with glucocorticoids (methylprednisolone) for 64 (range, 54–81) days, with a total dosage of 4162 (range, 2220–6060) mg.

The 22 subjects of the control group included 16 males (73%) and 6 females (27%), aged between 19 and 37 years, and ethnically comprised 19 Han Chinese and 3 minorities (1 Dong and 2 Tujia). The duration of exposure to TCE ranged from 13 to 147 weeks (MP_25_, 16, MP_75_, 56 weeks), and the concentration of TCE at the work environment was 14.6 (MP_25_, 10.7, MP_75_, 137.7 mg/m^3^, range, 5.4–655.6 mg/m^3^).

The 20 subjects of the validation group consisted of 10 males (50%) and 10 females (50%) aged between 17 to 51 years, and ethnically included 18 Han Chinese, one Zhuang and one Tujia. The duration of exposure to TCE ranged from 1 to 4 weeks in 14 (64%) cases, 5 to 8 weeks in 4 cases, and 9 to 11 weeks in 2 cases. The concentration of TCE at the work environment was 41.9 (MP_25_, 12.0, MP_75_, 78.0 mg/m^3^, range, 9.3–353.5 mg/m^3^).

There were no significant differences in age, gender, race and ethnicity, and concentration of TCE at the work environment among the three groups. However, there were significant difference in the exposure time to TCE (H = 43.589, *P* < 0.001).

### Results of the skin patch test

**Fig 1:**
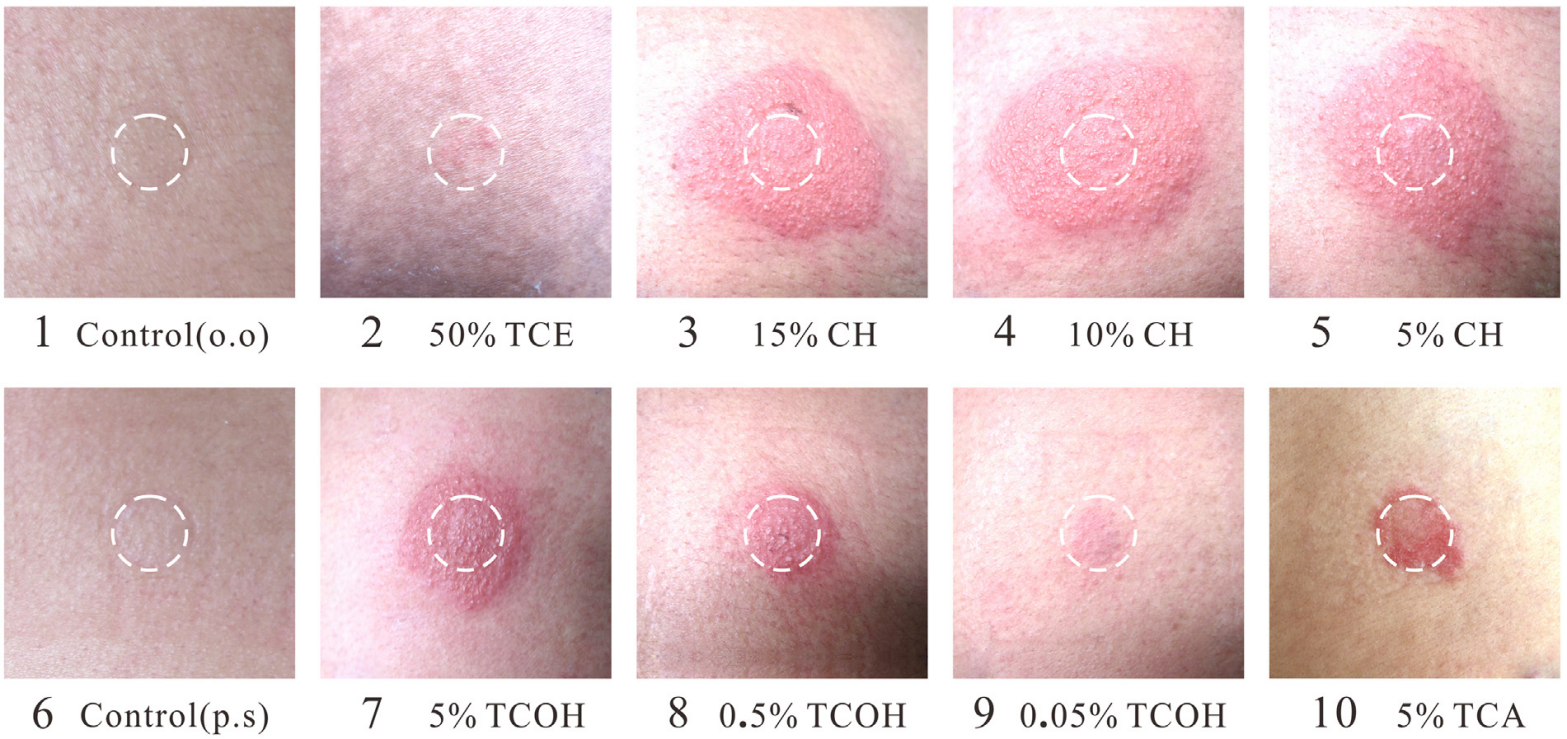
Results of the skin patch test. The white circle represents the circumference of the patch. A positive reaction (++) according to the ICDRG scoring system was observed for chloral hydrate (15, 10 and 5% in olive oil) and trichloroethanol (5% in olive oil). A weak positive reaction (+) was observed for TCE (50%), trichloroethanol (0.5 and 0.05% in olive oil) and trichloroacetic acid (5% in physiological saline). The reactions for TCE at each concentration (25, 10 and 5% in olive oil) and controls were negative.

None of the 61 subjects developed adverse symptoms during and after the skin patch test. Furthermore, none of the subjects showed reaction to olive oil, normal saline or adhesive tape. All 19 subjects with THS who had received treatment developed skin reactions the allergens used in the patch test, with the highest positive rate for CH (the positive rate for CH was 100% for 15%, 10% and 5% concentrations), followed by TCOH, TCA and TCE (Table 1). The lowest positive rate was for TCE, with a rate of 10.5% for 50% concentration and 0% for 25%, 10% and 5% concentrations (Table 1 and Fig 1). In contrast, the skin patch test was negative in all subjects of the control group, including CH, TCOH, TCA and TCE at the tested concentrations.

**Table 1:** Results of the skin patch test among 19 convalescents with THS.

Materials of patch test	n	Positive result (n)	Positive rate (%)
Physiological saline	19	0	0
Olive Oil	19	0	0
CH			
15%	19	19	100
10%	19	19	100
5%	19	19	100
TCOH			
5%	19	17	89.5
0.5%	19	14	73.7
0.05%	19	10	52.6
TCA			
5%	19	9	47.4
0.5%	19	0	0
TCE			
50%	19	2	10.5
25%	19	0	0
10%	19	0	0
5%	19	0	0

*χ* ^*2*^ = 196.799, *P*<0.001. CH: chloral hydrate in olive oil, TCOH: trichloroethanol in olive oil, TCE: trichloroethylene in olive oil, TCA: trichloroacetic acid in physiological saline.

Based on the positive rates and concentration, 15.0% CH solution was selected to test subjects of the validation group. The results of the patch test using CH at 15% were negative in all the subjects of the validation group. Subjects of the validation group did not develop THS during the follow-up period of 12 weeks.

Comparison of subjects of the case group who were positive and negative to TCOH and TCA showed no significant differences in gender, age, race and ethnicity, skin rash phenotype, duration of TCE exposure, urinary TCA level, initial dose of glucocorticoid, time of initiation and duration of glucocorticoid treatment.

## Discussion

TCE is a small molecule with a molecular weight of 131.39, making it difficult to trigger hypersensitivity reaction by itself [18,19]. In the present study, we hypothesized that the culprit compound responsible for THS is not TCE itself, but rather its metabolites. TCE is metabolized through two major pathways, cytochrome P450 (CYP)-dependent oxidation and conjugation with glutathione (GSH). The P450 pathway is a higher activity and higher affinity pathway than the GSH conjugation pathway [20]. Key CYP-derived metabolites of TCE include chloral (chloral reacts rapidly with water to form CH), CH, TCOH, TCA and dichloroacetic acid [15,20]. In the present study, TCE and its three main metabolites were used as allergens, and the skin patch test was conducted in THS-treated workers as well as healthy workers (exposed to TCE for more than 12 weeks but did not develop THS). Since the patch test using the four allergens showed positive results in the former group, while all the control subjects showed negative results, it is conceivable that TCE and its metabolites are involved in the pathogenesis of THS.

Previous *in vivo* study showed that CH and TCOH can transform into each other through enzymatic reactions [15]. The present study showed that the rates of positive patch test for CH were all 100% (19/19) for 15%, 10% and 5% concentrations, while the rates of positive patch test for TCOH were dose-dependent [89.5% (17/19), 73.7% (14/19) and 52.6% (10/19) for 5%, 0.5% and 0.05% concentrations, respectively]. These results suggest that both CH and TCOH play a pathological role in THS. However, considering that CH induced a positive skin reaction in every individual, it is the most likely candidate allergen responsible for the induction of occupational THS. In this regard, drug-induced lymphocyte stimulation test carried out by Watanabe et al [6] using CH showed a significantly high stimulation index (192 SI%) in a THS case compared with the normal value of 180 SI%. Those results add further support to our conclusion.

To validate the above conclusion, we tested new workers that had been exposed to TCE within 12 weeks using the skin patch test that included only 15% CH. The results showed that all 20 subjects of the validation group who did not develop THS had negative patch test and did not develop THS during the 12-week follow-up period. Taken together, the present study suggests that CH patch test could be potentially useful to identify subjects at high risk of development of THS among TCE-exposed workers.

The skin patch test is an important and simple test to confirm the diagnosis of allergic contact dermatitis and identification of the causative allergen(s), as well as identification of type IV (T cell-mediated or delayed) hypersensitivity reaction [21–23]. The present study showed that all subjects of the case group had positive patch test for TCE, suggesting that THS is induced following exposure to TCE and that the type of immune reactions of THS is mainly type IV hypersensitivity reaction. Our previous study showed high percentages of CD3^+^ and CD8^+^ T cells and significantly low CD4^+^ T cell count in patients with THS, compared with the Chinese reference values [24]. These results support the conclusion that type IV hypersensitivity reaction is the main immune reaction of THS. The results of the present study are in agreement with other studies that used the patch test in five cases with THS [6,11,13,16,25]. Considering the pathogenic role of IL-1β, IL-6, IL-8 and TNF-α in the inflammatory reactions of hypersensitivity dermatitis [26,27], Jia Q et al [28] found significantly higher levels of IL-1β, IL-6, IL-8 and TNF-α in THS patients than TCE-exposed workers and non-exposed controls. To elucidate the integral role of TCOH in the development of THS, the above study also examined the effects of TCE metabolites on IL-1β, IL-6, IL-8 and TNF-α expression *in vitro*. Considering that CH and TCOH can transform into each other by enzymatic reactions [15], the above findings add further support to the main conclusion that type IV hypersensitivity reaction is the main cause of THS. The results also highlight the utility of the patch test as a useful tool to determine the allergen responsible for occupational hypersensitivity diseases induced by certain chemicals.

The present study has several limitations. First, the incidence of THS was too low among the workers. Second, none of the subjects of the validation group showed a positive patch test and the sample size was too small to allow firm conclusions. For these reasons, we could not analyze the relation between a positive CH patch test before exposure to TCE and the onset of THS in the present study. Third, the study did not provide the reason for the negative TCOH and TCA patch tests in some subjects of the case group. Based on ethical considerations, we could not take skin biopsy samples from the patch test area to measure the CD4^+^ and/or CD8^+^ T cell infiltration. Further studies involving large sample size of THS cases and TCE-exposed subjects are needed to determine the molecular mechanism of THS and identify the culprit compound in TCE-induced THS.

## Conclusion

The present study based on the patch skin test with TCE and its metabolites in 19 subjects with THS suggested that exposure to TCE is the main cause of THS, and that the category of immune reaction of THS is mainly type IV hypersensitivity. THS is potentially mediated through its metabolite CH. The CH patch test could be potentially useful for the diagnosis of THS and identification of subjects at risk of THS.

## Supporting Information

S1 Text English translation of the Chinese National Diagnostic Criteria for Occupational Medicamentosa-like Dermatitis Induced by trichloroethylene. Trichloroethylene hypersensitivity syndrome (THS) is also called Occupational Medicamentosa-like Dermatitis Induced by Trichloroethylene according to the Chinese national legal occupational disease list. In China, every occupational disease should be diagnosed according to the national diagnostic criteria. Therefore, The Ministry of Health of the People’s Republic of China drafted and published the Chinese National Diagnostic Criteria for Occupational Medicamentosa-like Dermatitis Induced by trichloroethylene. The criteria consist of diagnostic principles, diagnostic criteria and specific clinical features for the skin disorder.
(DOC)

S1 Fig Representative rash phenotypes seen in THS. (a) and (b) Exfoliative dermatitis (ED) type. Physical examination showed dark erythematous skin lesions over the majority of the body with some confluence and scaling. (c) and (d) Erythema multiforme (EM) type. Eruption-type erythema, papules, and purpuric macules with pale erythematous outer ring, were observed on the entire body.
(TIF)
