# Supplementary material for: Trichloroethylene Hypersensitivity Syndrome Is Potentially Mediated through Its Metabolite Chloral Hydrate
Source: PLoS One. 2015 May 28;10(5):e0127101. doi: 10.1371/journal.pone.0127101 (PMC4447350; doi:10.1371/journal.pone.0127101)
Supplement: S1 Text — Trichloroethylene hypersensitivity syndrome (THS) is also called Occupational Medicamentosa-like Dermatitis Induced by Trichloroethylene according to the Chinese national legal occupational disease list. In China, every occupational disease should be diagnosed according to the national diagnostic criteria. Therefore, The Ministry of Health of the People’s Republic of China drafted and published the Chinese National Diagnostic Criteria for Occupational Medicamentosa-like Dermatitis Induced by trichloroethylene. The criteria consist of diagnostic principles, diagnostic criteria and specific clinical features for the skin disorder. (DOC) [file pone.0127101.s001.doc]

**S1. English translation of the Diagnostic Criteria of Occupational Medicamentosa-like Dermatitis induced by Trichloroethylene**

**1. Principles of diagnosis**

The diagnosis of occupational medicamentosa-like dermatitis induced by trichloroethylene requires comprehensive analysis of occupational exposure history to trichloroethylene, clinical manifestations of mainly acute skin inflammation, fever, liver damage and superficial lymphadenopathy, positive results of laboratory tests and occupational survey. On the other hand, similar hypersensitivity syndromes induced by other unequivocal reasons have to be excluded.

**2. Diagnostic criteria**

Skin damage manifests as acute dermatitis, mostly exfoliative dermatitis, followed by erythema multiforme, Stevens-Johnson syndrome or toxic epidermal necrolysis. The specific clinical features of these dermatological disorders are described in detail in section 3. Skin damage is often accompanied by fever, liver damage and superficial lymphadenopathy. The diagnostic criteria include:

2.1. A clear history of occupational exposure to trichloroethylene.

2.2. An incubation period ranging from 5 to 40 days, but often no more than 80 days.

2.3. Appearance of hypersensitivity syndrome among a group of workers but not the entire workforce under the same working conditions or doing the same type of work.

**3. Clinical features of dermatological manifestations**

3.1. *Exfoliative dermatitis:* The condition initially starts symmetrically as a diffuse erythematous maculopapular rash, which subsequently spreads to the entire body within several days, sometimes accompanied by swelling and partial coalescence. In severe cases, the rash develops into generalized edematous erythema, with conspicuous facial swelling, accompanied by exudates and incrustation. The rash becomes dark in color with increasing desquamation. The size of the scales could vary from pityroid to lamellar, and may be thick on the palm, which can exfoliate like torn gloves. The involved skin becomes dry and tight, and the patient may develop rhagadia, exudates and secondary infection affecting the angulus oris, neck, joints and chest. Reappearance of the rash with subsequent exfoliation occurs in some patients.

3.2 *Erythema multiforme:* In this phenotype, the skin lesions manifest erythema, papules and bullae. A typical erythema multiforme displays dark-red or purpuric macules with a pale erythematous outer ring, with or without subepidermal blisters in the center. In general, the mucous membrane, excluding the oral cavity, is spared.

3.3. *Stevens-Johnson syndrome:* Severe bullous multiform erythema involving oral, ocular and genital membranes.

3.4. *Toxic epidermal necrolysis:* The rash initially appears as bright red or reddish purple macules, which increase and expand rapidly, coalescing into larger dark reddish-brown lesions. Involvement of the entire skin and mucous membranes of various body cavities is noted in severe cases. Large and flaccid bullae rapidly appear on the rash, developing into generalized, widespread, and more or less symmetrical epidermal necrolysis, which forms a large number of parallel or zonal wrinkles measuring 3 to 10 cm in size. Nikolsky’s sign is positive, but purulence is rarely observed. Exfoliation of the ophthalmic, nasal and oral mucous membranes can also be observed in some cases.
