# Supplementary figures and images for: Trichloroethylene Hypersensitivity Syndrome Is Potentially Mediated through Its Metabolite Chloral Hydrate
Source: PLoS One. 2015 May 28;10(5):e0127101. doi: 10.1371/journal.pone.0127101 (PMC4447350; doi:10.1371/journal.pone.0127101)

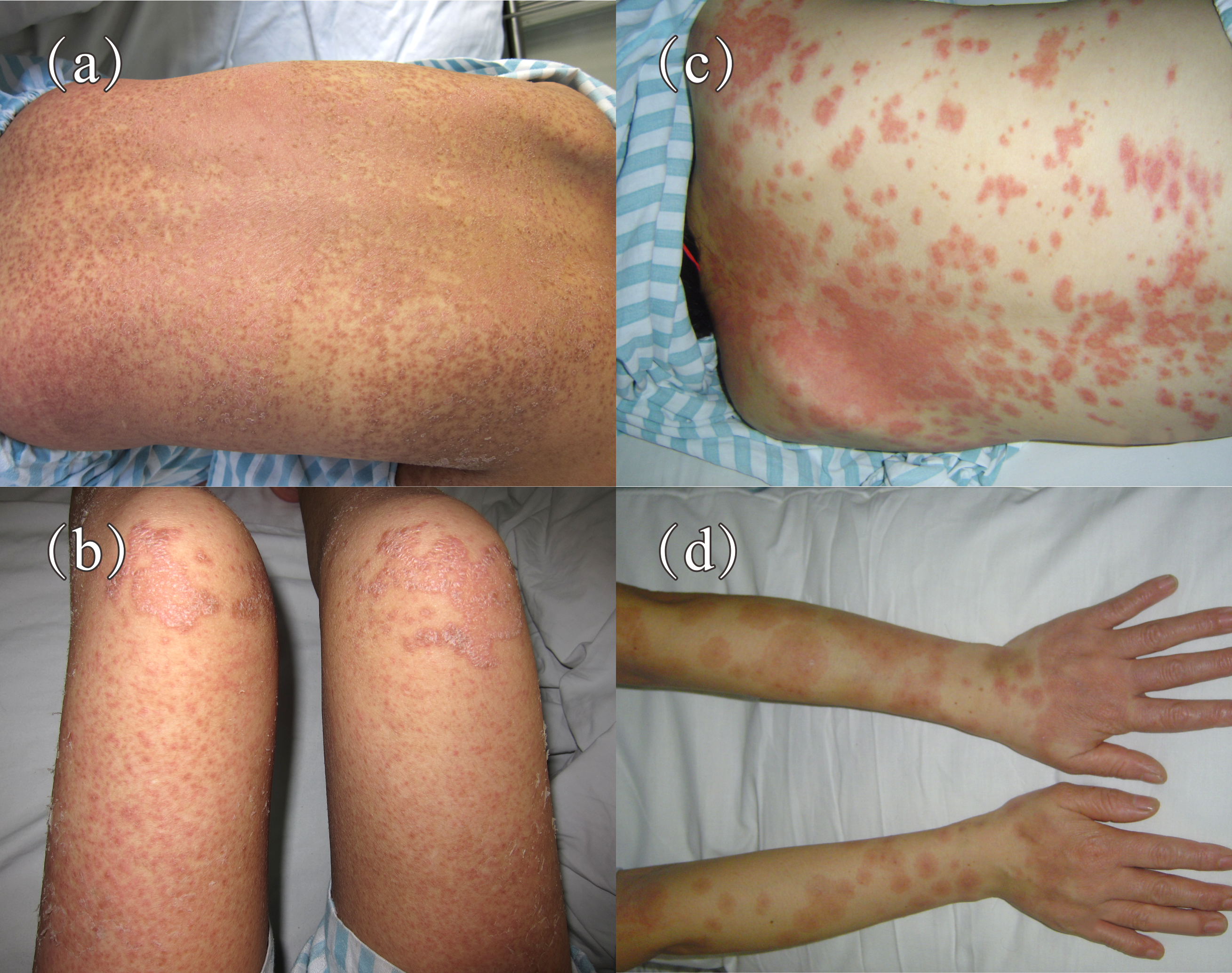

Supplement: S1 Fig — (a) and (b) Exfoliative dermatitis (ED) type. Physical examination showed dark erythematous skin lesions over the majority of the body with some confluence and scaling. (c) and (d) Erythema multiforme (EM) type. Eruption-type erythema, papules, and purpuric macules with pale erythematous outer ring, were observed on the entire body. (TIF) [file pone.0127101.s002.tif]
